# Editorial: Metabolic reprogramming for acquiring therapeutic resistance in glioblastoma

**DOI:** 10.3389/fonc.2023.1220063

**Published:** 2023-07-14

**Authors:** Tsung-I Hsu

**Affiliations:** ^1^ Ph.D. Program in Medical Neuroscience, College of Medical Science and Technology, Taipei Medical University and National Health Research Institutes, Taipei, Taiwan; ^2^ International Master Program in Medical Neuroscience, College of Medical Science and Technology, Taipei Medical University, Taipei, Taiwan; ^3^ Taipei Medical University Research Center of Neuroscience, Taipei, Taiwan; ^4^ Taipei Medical University Research Center of Cancer Translational Medicine, Taipei, Taiwan

**Keywords:** metabolic reprogramming, therapeutic resistance, glioblastoma, temozolomide, mass spectrometry, orexin A, flavins, succinylation

This Research Topic is very popular and important in cancer research, especially in the treatment of glioblastoma (GBM), a very aggressive malignant brain tumor. Despite efforts to understand mechanisms of treatment resistance in GBM, the high mortality associated with this disease remains a major challenge. One of the main factors contributing to this problem is resistance to chemotherapy, including the first-line chemotherapy drug temozolomide (TMZ) for GBM. One of the main ways cancer cells evade the effects of chemotherapy is through metabolic reprogramming, which involves changes in cellular metabolism to adapt to the cellular stress induced by chemotherapy. This reprogramming allows GBM cells to survive and continue to grow, leading to treatment resistance. Understanding the mechanisms of metabolic reprogramming and how it contributes to the development of resistance to GBM therapy is an active area of research and holds great promise for the development of new and more effective therapies for the disease.

An unmet need in the field is the lack of a comprehensive understanding of the metabolic changes that occur in GBM cells in response to chemotherapy and how these changes lead to treatment resistance. In addition, there is a need to develop new and innovative approaches to address the metabolic changes that lead to resistance to GBM therapy. This includes identifying new drug targets, developing new drugs, and establishing new therapeutic strategies that effectively target the metabolic changes that occur in GBM cells.

Reichert et al. developed a method that incorporates the use of fluorescence lifetimes and autofluorescent optical redox ratios of flavins, which are metabolic enzymes involved in redox processes. By measuring the fluorescent properties of these enzymes, scientists can gain information about the metabolic state of brain tumors and potentially identify markers of treatment resistance. This information can be used to develop new treatments for GBM. The use of fluorescence lifetime and the autofluorescent optical redox ratio to improve visualization of brain tumors is a novel approach that may have significant advantages over traditional diagnostic methods. The fluorescence lifetimes of naturally occurring flavins *in vivo* can provide valuable information about the metabolic state of cells. Autofluorescent optical redox ratios are also powerful diagnostic tools, as they can provide information about the oxidative state of cells, which is important for understanding the basic biology of brain tumors.

Mass spectrometry (MS)-based proteomics has made significant progress in recent years and has great potential to help understand the molecular drivers of GBM and TMZ resistance. Teraiya et al. discussed the potential benefits and insights of using global proteomics technologies to further understand mechanisms of drug resistance at the molecular level. By analyzing a large number of proteomic samples from different laboratories, biomarkers can be confidently identified and new efforts to understand TMZ resistance can be made. The creation of an international LC-MS proteomics database of TMZ-resistant cells would be an important step in this direction and could lead to the identification of new targets and the design of new drugs, including personalized medicine. Studying a large number of TMZ-resistant tumors using liquid chromatography (LC)-MS-based proteomics/metabolomics can reveal mechanisms or pathways underlying the development of drug-resistant phenotypes, which are critical for the development of personalized therapies and new therapeutic agents.

The study of orexin A by Yang et al. involves the use of multi-omics integration, the integration of multiple types of omics data, to gain a comprehensive understanding of the biological system under study. Regarding orexin A and GBM, the authors investigated the effect of orexin A on GBM cells using transcriptomic, proteomic, and metabolomic data. The authors found that orexin A was able to induce metabolic reprogramming in GBM cells, resulting in changes in the expression of various genes and metabolic proteins, as well as changes in the metabolic profile of the cells. The results of this study suggest that orexin A may play a role in the development of resistance to GBM therapy. By inducing metabolic reprogramming, orexin A can help GBM cells avoid the effects of chemotherapeutic drugs and radiation therapy, which can lead to the development of drug resistance. This is an important finding as it provides new insights into the molecular mechanisms underlying GBM therapy resistance and highlights the importance of studying the metabolic changes that occur in GBM cells. Furthermore, the use of multi-omics integration to study the effects of orexin A on GBM highlights the importance of systems biology approaches to study complex diseases such as GBM.

LC-MS is a powerful tool for analyzing post-translational modifications (PTMs) such as succinylation and determining the redox state of cancer cells. Succinylation is a PTM, the covalent modification of lysine residues in proteins by the addition of succinyl groups. It has been shown to play a role in regulating various cellular processes, including cellular metabolism and redox state, both of which are key factors in cancer development and progression. The integration of these two factors (succinylation and redox state) by Dai et al. provides valuable information on mechanisms of metabolic reprogramming and their impact on resistance to GBM therapy. By studying changes in succinylation and redox state in GBM cells, researchers can better understand the metabolic pathways that change in response to chemotherapy and how these changes contribute to the development of drug resistance. In particular, the study demonstrates that changes in succinylation and redox state contribute to the activation of specific signaling pathways that promote cell survival, thereby contributing to the development of therapeutic resistance. This information can be used to develop new strategies to target these pathways and overcome resistance. In addition, the study could reveal new therapeutic targets, such as specific enzymes involved in metabolic pathways that are altered by chemotherapy.

In conclusion, the study of metabolic reprogramming for acquiring therapeutic resistance in GBM is a rapidly growing field that has great potential for future development. Through a better understanding of the metabolic changes that lead to drug resistance, we may be able to develop more effective treatments for GBM and improve patient outcomes.

## Author contributions

The author confirms being the sole contributor of this work and has approved it for publication.

